# Homozygous Transgenic Barley (*Hordeum vulgare* L.) Plants by Anther Culture

**DOI:** 10.3390/plants9070918

**Published:** 2020-07-20

**Authors:** Ludmila Ohnoutková, Tomáš Vlčko

**Affiliations:** Laboratory of Growth Regulators, Palacký University & Institute of Experimental Botany, Czech Academy of Sciences, Šlechtitelů 241/27, 78371 Olomouc, Czech Republic; tomas.vlcko@upol.cz

**Keywords:** androgenesis barley, double haploid, embryo culture, transformation

## Abstract

Production of homozygous lines derived from transgenic plants is one of the important steps for phenotyping and genotyping transgenic progeny. The selection of homozygous plants is a tedious process that can be significantly shortened by androgenesis, cultivation of anthers, or isolated microspores. Doubled haploid (DH) production achieves complete homozygosity in one generation. We obtained transgenic homozygous DH lines from six different transgenic events by using anther culture. Anthers were isolated from T0 transgenic primary regenerants and cultivated in vitro. The ploidy level was determined in green regenerants. At least half of the 2n green plants were transgenic, and their progeny were shown to carry the transgene. The process of dihaploidization did not affect the expression of the transgene. Embryo cultures were used to reduce the time to seed of the next generation. The application of these methods enables rapid evaluation of transgenic lines for gene function studies and trait evaluation.

## 1. Introduction

The plant life cycle includes the switch between the haploid gametophytic phase and the diploid sporophyte phase. This change is considered so critical that it is not possible to interfere with it. Since 1964, when the development of haploid embryos and plantlets from microspores of *Datura inoxia* was performed using anther cultures, there has been extensive research on the process and its use. This process of inducing haploid or doubled haploid (DH) plants from meiotic spores (microspores) through anther culture or isolated microspores cultivated in vitro is called androgenesis [1]. Androgenesis is a form of apomixis in which the male gamete develops into embryos that can regenerate the whole plant.

The production of pure, homozygous plants is an important step in biological and genetic studies and in crop breeding. The homozygous lines are frequently based on several breeding programs, including mutation breeding and backcrossing. Moreover, DH plants facilitate studies in molecular genetics and genomics, such as genome mapping, quantitative trait loci (QTL) analysis, molecular marker-assistant selection, gene isolation, and cloning [2]. Production of DH plants is valuable for breeding programs, especially for self-pollinating cereal crops, such as barley. Pure lines can significantly reduce the time of the breeding process. Populations derived from pure lines allow the study of phenotypic traits of selected genotypes in different environmental conditions and developmental stages [3].

The androgenic progeny obtained from male meiotic spores (microspores) through the cultivation of anthers or isolated microspores can have a haploid or diploid nuclear constitution, with, exceptionally, tetraploid or mixoploid plants being obtained. Anthers are removed from flowers, preferably at the uninucleated stage, and either cultured directly under in vitro conditions, or microspores can be isolated prior to cultivation.

Stable genetic plant material is necessary for the exploration of gene function or for the breeding process. There are two main approaches to achieve this goal. Either a two-phase process consisting of plant transformation with subsequent development of homozygous plant material via haploid techniques, or a combination of the two steps by direct transformation of haploid plant tissues. Both of these approaches are feasible, although the latter strategy is more demanding, as manipulation with isolated haploid cells is technically more difficult under in vitro conditions and requires more practical experience.

The straightforward process is to prepare the transgenic material by using existing optimized plant transformation protocols. The identified transgenic plant then can be used for transgene stabilization. Either anthers [4] or isolated microspores [5] from T0 transgenic plants can be cultivated in vitro and regenerated into plants. Moreover, Kapusi et al. [6] reported that haploid techniques applied in the T0 generation after transformation could be effectively used for the elimination of the selectable marker gene.

To prepare homozygous transgenic material from haploid tissues, several methods of transformation have been developed. Without doubt, the transformation of haploid material represents a shortcut for obtaining homozygous plants. Anther cultures or haploid techniques have been used for several decades to obtain stable homozygous plants. In combination with plant transformation, it is a feasible way to accelerate the process. For the transformation, *Agrobacterium tumefaciens* or a particle bombardment are often used. The simplest way is co-cultivation of plant anthers with *Agrobacterium* suspensions, which has been reported to work efficiently for barley [7]. Similarly, not only barley anthers, but also ovules could be transformed by this method [8]. A more sophisticated method is transformation of isolated microspores. These methods require precise estimation of the developmental stage of the plant material. Apart from classical *Agrobacterium*-mediated transformation, electroporation as a means of transformation can be applied [9]. Notably, isolated microspore transformation proved to be very useful when trying to induce mutation using sequence-specific nucleases such as CRISPR/Cas9 [10]. Alternatively, anther calli derived from anthers or microspores could be used for transformation. Such transformation, however, requires optimization of the protocol, as limited calli could exhibit very low transformation efficiency [11,12]. Since T0 transgenics are heterozygous, there is a mixture of transgenic and non-transgenic T1 gametes, resulting in a mixture of homozygous transgenic and non-transgenic individuals. For identification of homozygous T1 transgenic plants, routine PCR screening could be performed.

**Figure 1:**
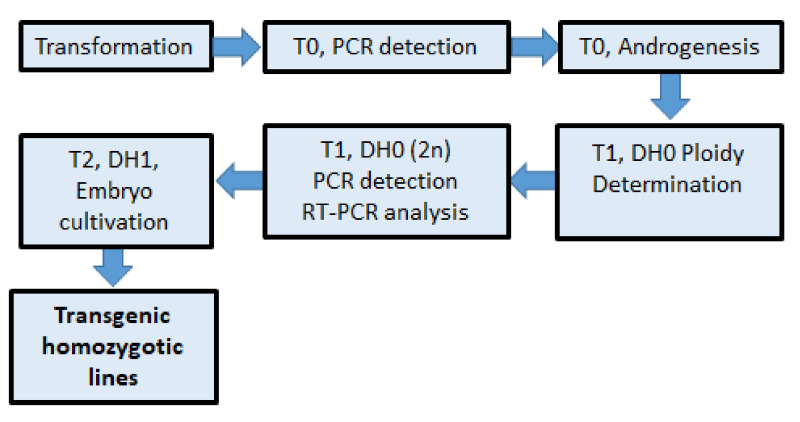
Scheme for rapid production of transgenic homozygous barley lines.

In the following work, a combination of plant transformation followed by anther cultures from T0 regenerants was performed to obtain homozygous progeny. Transgenic barley plants were evaluated for ploidy level, transgene heritability, and expression. This work summarizes the production of pure transgenic lines for use in molecular studies and crop breeding, as illustrated in Figure 1.

## 2. Results

### 2.1. Anther Cultures of Transgenic Plants

In our study, anthers from transgenic plants produced in the corresponding author’s laboratory were used to prepare doubled haploid plants by in vitro culture. In total, six different transgenic progenies were used to obtain pure homozygous lines. Verified transgenic plants were grown in the greenhouse until the optimal stage for spike collecting. Anthers were isolated after tetrad development (Figure S1a), when microspores were in the middle to late-uninucleate stage (Figure S1b)

Anthers of plants originating from each transformation experiment were collected, isolated, and cultivated in vitro on MN6 medium. The number of cultivated anthers differed for each transgene. After 4 weeks, embryogenic structures were observed, and two weeks later, regeneration started (Figure S2a). Regenerating plantlets were transferred onto the regeneration medium in flasks. Albino and green plants were observed (Figure S2b), with a high proportion of albino plants produced for all transgenes. For barley cultivar Golden Promise, the rate of regenerated green plants was in the range 1–4% (Table 1). Notably, approximately one third of regenerated plants from anther cultures with WDV (Wheat Dwarf Virus) Rep transgene were albino.

### 2.2. Ploidy Determination

**Figure 2:**
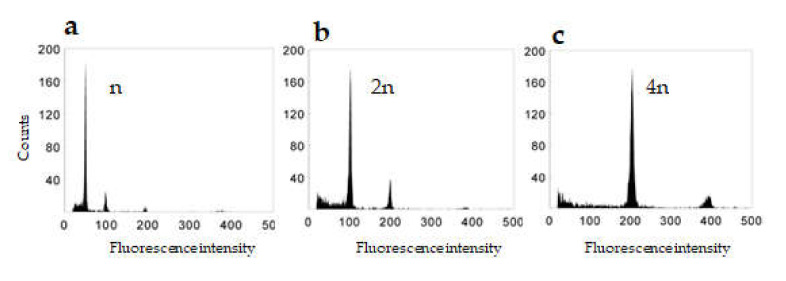
DNA content of nuclei isolated from leaves of transgenic plants regenerated from anthers (**a**) haploid plant (*n*), (**b**) dihaploid plant (2n), (**c**) tetrahaploid plant (4n).

In our work, flow cytometry was used to determine the ploidy level in regenerated green plants (Figure 2), revealing that haploid and spontaneous doubled haploid plants were obtained in a ratio of approximately 1:1 (Table 1). Spontaneous tetraploid and aneuploid genotypes were also obtained. However, these regenerated at very low frequencies and were not present for all transgenes. Indeed, aneuploids were identified for only the *Lim*, *Bar*, and *Osm* transgenes. Transgenic plants that exhibited the DH 2n phenotype accounted for roughly three-fifths of all regenerated 2n plants independently of the transgene, i.e., transformation experiment. Until the tillering stage, there are no visible phenotype differences between the n, 2n, and 4n genotypes. However, at the flowering stage, it is possible to distinguish phenotypic differences (Figure S3).

### 2.3. Genotyping

**Figure 3:**
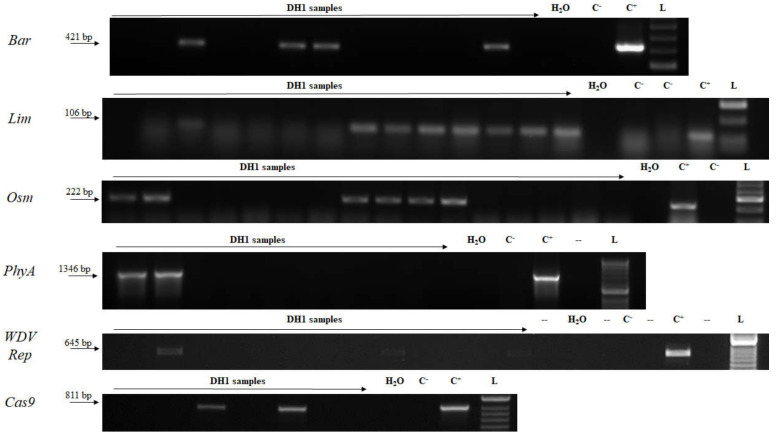
Detection of the transgenes in anther culture regenerants. L—DNA ladder (HyperLadder 50-bp, Bioline); C^+^—genomic DNA from T0 transgenic plant; C^−^—wild-type plant; DH1—samples of anther culture regenerants. *Bar*—barnase; *Lim*—limen; *Osm*—osmotin; *PhyA*—phytase; *WDV Rep*—replication protein gene; *Cas9*—CRISPR associated protein 9.

Regenerated green plants from anther cultures were analyzed for the presence of the transgene. Specific amplicons for each transgene (*Bar, Lim, Osm, PhyA, WDV Rep, Cas9*) were detected in populations of DH1 regenerants by PCR (Figure 3). In total, generally more than half DH green regenerated plants proved to be transgenic (Table 1).

### 2.4. Transcript Detection

**Figure 4:**
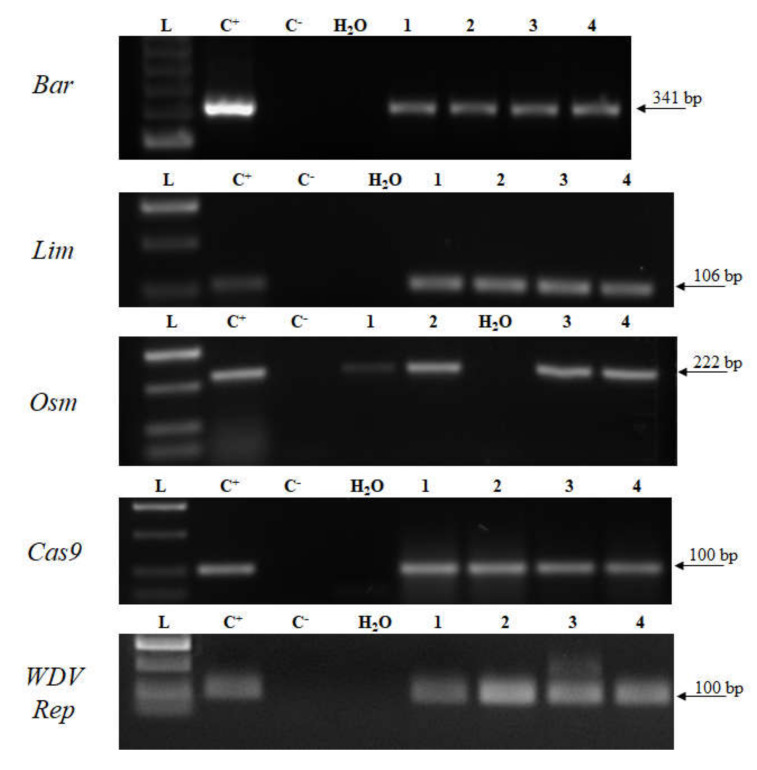
Detection of transcripts in selected DH1 transgenic plants. L—DNA ladder (HyperLadder 50-bp, Bioline); C^+^—plasmid DNA or transgenic plant; C^–^—wild-type cDNA sample; 1–4—cDNA samples of DH1 transgenic plants. *Bar*—barnase; *Lim*—limen; *Osm*—osmotin; *WDV Rep*—replication protein gene; *Cas9*—CRISPR associated protein 9.

**Figure 5:**
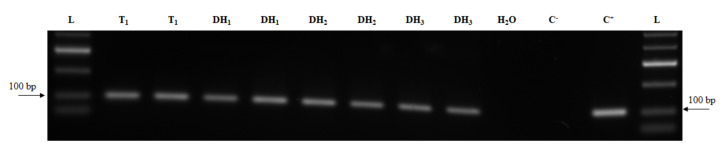
Detection of *Cas9* transcript over three successive DH generations. L—DNA ladder (HyperLadder 50-bp, Bioline); C^+^—plasmid DNA; C^–^—wild-type cDNA sample; T_1_ and DHs—cDNA samples of transgenic line and DHs.

Analysis of transgene expression is important for evaluation of transgene activity and it is necessary for gene function studies. Here, transgene transcription was analyzed in DH1 generation and the transcripts were detected for all studied transgenes (Figure 4). To further study *Cas9* transgene expression, *Cas9* transcripts were evaluated over three generations from primary T0 regenerants to DH1 and DH2 homozygous plants. As shown in Figure 5, transcripts of *Cas9* were identified in all three generations.

### 2.5. Embryo Cultures

**Figure 6:**
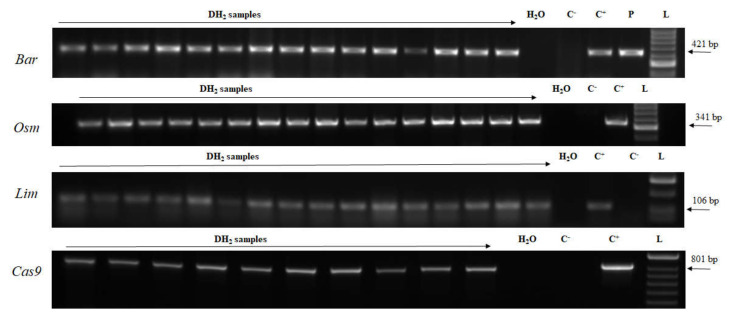
Detection of the transgenes in DH2 generation transgenic plants. L—DNA ladder (HyperLadder 50-bp, Bioline); C^+^—genomic DNA sample of T_0_ transgenic plant; P—plasmid DNA; C^–^—wild-type plant; DH2 samples—genomic DNA samples. *Bar*—barnase; *Lim*—limen; *Osm*—osmotin; *PhyA*—phytase; *WDV Rep*—replication protein gene; *Cas9*—CRISPR associated protein 9.

Seed maturation and subsequent seed dormancy is a natural plant process requiring a lot of time. By means of young embryo cultures under in vitro conditions (Figure S4), the time required for seed maturation was reduced in our experiments and a population of transgenic DH plants were hastened. In order to confirm the stability of the transgene in the DH plants, progenies of selected plants were screened. Accordingly, consistent with stable genetic material, the transgene was confirmed in the genomic DNA of all the analyzed plants (Figure 6). Healthy plants were transferred into pots and grown in the greenhouse for phenotype evaluation and harvesting (Figure S5).

## 3. Discussion

Transgenic homozygous lines are a desirable prerequisite for phenotyping and subsequent trait evaluation. Previous studies reported that there are two feasible strategies for rapid development of transgenic homozygous lines. Either haploid cells, microspores, and ovules are transformed [7,8], or microspores and anthers of transgenic plants are cultivated after transformation [4,5,6]. However, technically, a simpler method is cultivation of anthers collected from the transgenic plants rather than microspore cultures. Many studies reported that androgenesis has a high potential for biological and molecular studies, as well as for plant breeding of crop plants [13,14,15,16]. Conventional selection protocols for obtaining pure homozygous transgenic lines is approximately 10–11 years in the monocot crop species, barley and wheat. DH technology, which has an advantage in achieving complete homozygosity in one generation, is a major improvement on classical approaches by substantially shortening the time to obtaining pure lines.

Androgenesis consist of two steps: (i) induction of pollen embryogenic structure or calli, (ii) plant regeneration. The main factors affecting the process of DH plant production are genotype and physiological condition of the donor plants, developmental stage of the gametes, pre-treatment, culture medium, and physical factors during tissue culture [17]. The efficiency of androgenesis is determined by the ability to induce embryogenic callus, the number of regenerated plants, the proportion of regenerated green and albino plants, the ploidy level of green plants, and the number of spontaneous DH plants.

Increased numbers of regenerated doubled plants can be achieved by treatment with chemical reagents such as colchicine, which induce diploidization in haploid material [18,19]. In contrast, our protocol [20] allows sufficient numbers of DH regenerants to be obtained by spontaneous diploidization without colchicine treatment. Spontaneous DHs were obtained also for other plant species, such as *Brassica napus* [21].

In our results, a high proportion of the regenerated plants were albinos. Such a phenomenon is not normally observed in in vitro cultures from immature embryos for plant transformation. In anther cultures, a higher number of regenerating albino plants in comparison to green plants has been observed [5,22]. Indeed, in a test of 20 spring barley cultivars, the mean portion of regenerated albino plants reached over 90% [23]. Albinism is a major constraint in the production of DH plants. Sriskandarajah et al. [24] increased the proportion of green plants of spring barley, cultivar Mitja, from 6 to 42% by altering the culture method and composition of the culture medium. In a new three-step protocol, the addition of casein hydrolysate to an intermediate regeneration medium gave the highest number of green plants. Unraveling the potential cause of albino plant development, Gajecka et al. [25] suggested that green plant regeneration depends on the state of plastid differentiation in the microspores at the stage of culture initiation.

In our study, the largest proportion of albino plants, 27.8%, was observed after transformation with the WDV Rep transgene (Table 1). Interestingly, a larger proportion of albino plants was also observed in the primary regenerants obtained after transformation with this WDV-derived transgene, which is derived from a common barley and wheat pathogen [26]. Hence, increased regeneration of albino plants, in this case, can be accounted for by the residual activity of the truncated replication protein rather than the developmental stage of the pollen grain. Based on our results collected from anther cultures of several transformation experiments, it can be concluded that there is no direct effect of a transgene on the regeneration capacity of the selected genotype, provided that the gene product does not interfere with the basic cell processes, such as meiosis, etc. Since albino plants do not survive the transfer from in vitro to in vivo conditions, they were not further analyzed.

Doubling of the haploid genome doubles the DNA content in the nucleus. Different methods can be used to assess the ploidy level [27]. The simplest method to assess the ploidy level through the analysis of cellular DNA content is flow cytometry, which is a very fast and reliable method. In our work, ploidy levels were in accordance with published results [24]. Transgenic plants can suffer from transgene silencing. This phenomenon was described as a reaction by a host organism to the presence of a foreign genetic element. A higher copy number of a transgene can even increase the probability of gene silencing [28,29]. In this regard, the production of doubled haploids can induce increased transgene expression, which in turn may attract cell control machinery and result in gene silencing. Melander et al. [22] studied transgene expression over five generations in DH plants, reporting that gene silencing occurred in one individual. No signs of gene silencing were detected for any of the transgenes used in our work, including *Cas9*, which was analyzed over three DH generations.

In our experiment we included young embryo cultures to reduce the time needed for one generation in barley. Yan et al. [3] reported that embryo cultures can greatly reduce the generation time in plant breeding. Embryo cultures circumvent the need for mature seed development, the dormancy phase, and possibly vernalization in winter genotypes. We used embryo cultures to rapidly obtain the next generation of DH plants, and this step can be repeated in further generations.

Spring barley cv. Golden Promise and winter barley cv. Igri are considered model barley cultivars for transformation and anther culture, as they are known for their high androgenic activity. Spontaneous DH plants obtained from the transformation experiments were completely fertile. It is not necessary to include the colchicine treatment for dihaploidization during the androgenesis process, as spontaneous dihaploidization provides sufficient 2n plants. Gene expression analysis showed that androgenesis did not affect transgene expression in either of the studied transgenes. Moreover, analysis of three generations of Cas9 DHs confirmed transgene expression stability. This paper is the first to give full information about how to obtain homozygous lines from transgenic lines simply and rapidly by this combination of two in vitro methods.

## 4. Materials and Methods

### 4.1. Plant Material

Transgenic plant material was obtained from the transformation experiments conducted in the authors’ laboratory. The development of Osmotin transgenic plants has been described elsewhere [30]. Limen transgenic plants were reported by Rehorova et al. [31] and partial WDV replication protein gene-expressing plants were described in work by Cejnar et al. [26]. Cultivar Golden Promise was used for each transformation event. The transgenes were *Bar*—barnase; *Lim*—limen; *Osm*—osmotin; *PhyA*—phytase; *WDV Rep*—replication protein gene; and *Cas9*—CRISPR associated protein 9.

### 4.2. Anther Cultures

Anthers were isolated from the transgenic T0 plants and cultivated in vitro according to the protocol by Ohnoutkova et al. [20].

### 4.3. Detection of Transgenic Plants

PCR screening of T0 primary regenerants or plants from anther cultures was performed using genomic DNA extracted from young plant leaves, according to the protocol by Edwards et al. [32]. In the reaction, REDTaq^®^ ReadyMixTM PCR Reaction Mix (Sigma-Aldrich, USA) was used. For a list of primers used for each gene (Table 2). PCR was performed in 10 μL volumes consisting of 5 μL ReadyMix, 2.5 μL nuclease-free water, 0.25 μL 10 μM of each primer, and 2 μL of sample genomic DNA. Reaction conditions were the same for each reaction, except annealing temperature, which differed depending on the primer pairs. Cycling conditions were 94 °C for 3 min, then 38 cycles of 30 s at 94 °C, and 30 s annealing at the temperatures listed in Table 2, with elongation for 60 s at 72 °C. PCR products were separated on 1% agarose gel stained with ethidium bromide.

### 4.4. Ploidy Level

DH transgenic plants were evaluated for ploidy level using flow cytometry as described by Dolezel and Göhde [33].

### 4.5. Detection of Transcripts

If not stated otherwise, all procedures were performed according to the manufacturer’s conditions. For transcript detection, total RNA from young leaves was extracted using TriReagent (Sigma-Aldrich). RNA was dissolved in PCR-grade water and stored at 4 °C for one hour. Then, the RNA concentration was determined spectrophotometrically and the quality of RNA was verified using denaturing gel electrophoresis [34]. RNA samples were treated with Turbo DNase (ThermoFisher). A total of 1 μg of total RNA was used to reverse transcribe with RevertAid H minus Reverse Transcriptase (ThermoFisher) and poly-T primers. The resulting cDNA was 10-fold diluted with water and stored at −20 °C till analysis. REDTaq^®^ ReadyMixTM PCR Reaction Mix (Sigma-Aldrich, USA) and primers listed in Table 3 were used for PCR as described above. Primers for Bar and Lim genes were as used for the genotyping. The PCR conditions were initial denaturation for 3 min at 94 °C, then 40 cycles of 15 s at 94 °C, 20 s at 60 °C, and 30 s at 72 °C.

### 4.6. Embryo Culture

Developing grains from the transgenic plants, 21 to 28 days after anthesis (embryo size in range 1.5–2 mm), were collected in a flask and surface sterilized in 70% ethanol for 1 min, followed by washing in 1% sodium hypochlorite for 10 min. The grains were then rinsed three times with sterile water. Embryos were dissected from caryopsis under aseptic conditions and placed on half-strength MS medium [35] containing 2% sucrose. Plates containing embryos were kept on a 16 h photoperiod in the phytotron at a light intensity of 300 μmol m^−2^ s^−1^ and a temperature of 20 °C. After a three-week cultivation, plantlets were transferred to soil and grown in the greenhouse.

## 5. Conclusions

In our study, we addressed the question of how to simply and quickly obtain homozygous lines from T0 transgenic barley plants. Based on our long-time experience with the preparation of transgenic plants, we show that it is possible to obtain stable transgenic material rapidly using a combination of the methods described above. We demonstrate that this protocol is reliable and homozygous transgenic DHs can be obtained within only two generations. The described procedure enables the fast study of genotype and phenotype of transgenic plants and can significantly help to introduce transgenic plants into practice.

## Figures and Tables

**Table 1:** Anther cultures from transgenic plants, showing numbers of green and albino plants, and ploidy level.

Transgene	No. of Cultivated Anthers	Regenerated Plants % (No.)		Ploidy Level Green Plants % (No.)				Transgenic Plants 2n % (No.)
		Green	Albino	n	2n	4n	Aneupl	
*Bar*	3547	1.3 (46)	5.2 (184)	39.1 (18)	52.2 (24)	6.5 (3)	2.1(1)	58.3 (14)
*Lim*	2428	1.3 (31)	4.4 (109)	38.7 (12)	45.2(14)	6.6 (2)	9.7(3)	50.0 (7)
*Osm*	1209	2.8 (35)	3.0 (36)	42.8 (15)	54.3 (19)	0	2.8 (1)	73.7 (14)
*PhyA*	612	2.6 (16)	5.3 (33)	50.0 (8)	43.8 (7)	6.3 (1)	0	85.7 (6)
*WDV Rep*	797	4.6 (37)	27.8 (222)	70.0 (26)	30.0 (11)	0	0	54.5 (6)
*Cas9*	1288	4.0 (52)	1.2 (15)	55.8 (29)	44.2 (23)	0	0	47.8 (11)

*Bar*—barnase; *Lim*—limen; *Osm*—osmotin; *PhyA*—phytase; *WDV Rep*—replication protein gene; *Cas9*—CRISPR associated protein 9. Aneupl—aneuploid.

**Table 2:** List of genotyping primers, amplicon sizes and annealing temperatures (TA).

Transgene	Primer Pair 5′–3′ Oriented	PCR Amplicon	TA [°C]
*Bar*	GGT CTG CAC CAT CGT CAA CC	421 bp	65
	GTC ATG CCA GTT CCC GTG CT		
*Lim*	AGC TTG GTA CCA CCA TGC AC	106 bp	57
	CAG TGG TCG TCG CAG TTA GA		
*Osm*	CTC CTC GAC GGC TTC AAC AT	341 bp	57
	TCG AGT GGG AAG TTT GGG TG		
	GCC CTG CCT TCA TAC GCT AT	222 bp	58
	TAC GGG CAG TTG TTC CTC AC		
*PhyA*	GGC AGT CCC CGC CTC GAG AAA	1346 bp	55
	AAA CAC TCC GCC CAA TCA CCC		
*WDV Rep*	ATGGCCTCTTCATCTGCACC	645 bp	63
	TGATTCGAGGCTTACGGAGT		
*Cas9*	TTC GCT ACT GTT CGC AAG GT	811 bp	58

**Table 3:** List of RT-PCR primers and amplicon sizes.

Transgene	Primer Pair 5′–3′ Oriented	PCR Amplicon
*Bar*	GGT CTG CAC CAT CGT CAA CC	421 bp
	GTC ATG CCA GTT CCC GTG CT	
*Lim*	AGC TTG GTA CCA CCA TGC AC	106 bp
	CAG TGG TCG TCG CAG TTA GA	
*Osm*	GCC CTG CCT TCA TAC GCT AT	222 bp
	TAC GGG CAG TTG TTC CTC AC	
*Cas9*	CGA CGC TAC TCT CAT CCA CC	100 bp
	CTT TTT GGT GGC AGC AGG AC	
*WDV Rep*	TTC ATC TGC ACC CAG GTT CC	100 bp
	GTG CGA AGT GAA TCC AAG GC	

## Supplementary Materials

The following are available online at https://www.mdpi.com/2223-7747/9/7/918/s1, Figure S1: Barley microspores stained by acetocarmine, Figure S2: Induction and regeneration processes in barley anther culture, Figure S3: Regenerated plants from anthers of transgenic barley T1 generation, Figure S4: Transgenic barley embryo culture, Figure S5: Transgenic plants (DH1 generation) regenerated from anther cultures.
