# Supplementary material for: Homozygous Transgenic Barley (Hordeum vulgare L.) Plants by Anther Culture
Source: Plants (Basel). 2020 Jul 20;9(7):918. doi: 10.3390/plants9070918 (PMC7412030; doi:10.3390/plants9070918)
Supplement: Supplementary file 1 [file plants-09-00918-s001.pdf]

Supplemental File

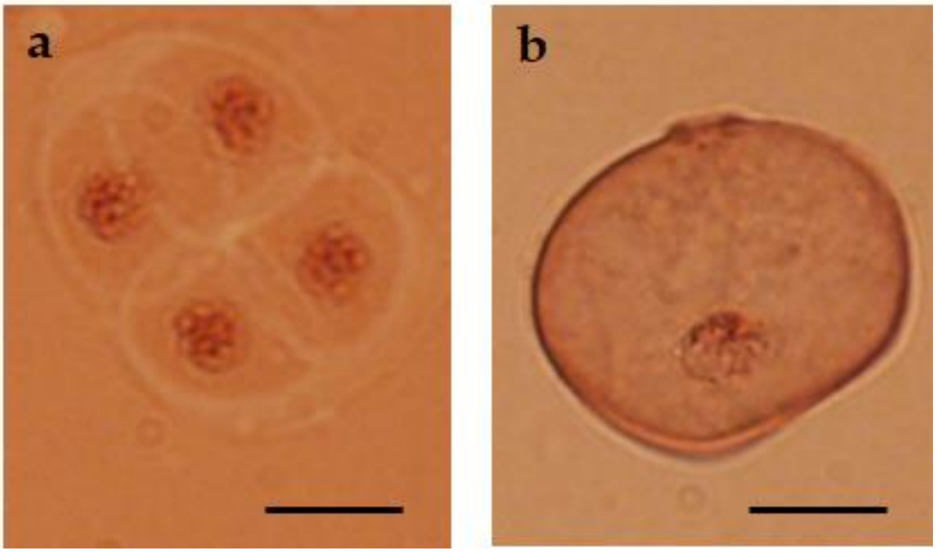

Figure S1. Barley microspores stained by acetocarmine, (**a**): microspore tetrad, (**b**): late-uninucleate microspore, Bar 10 μm.

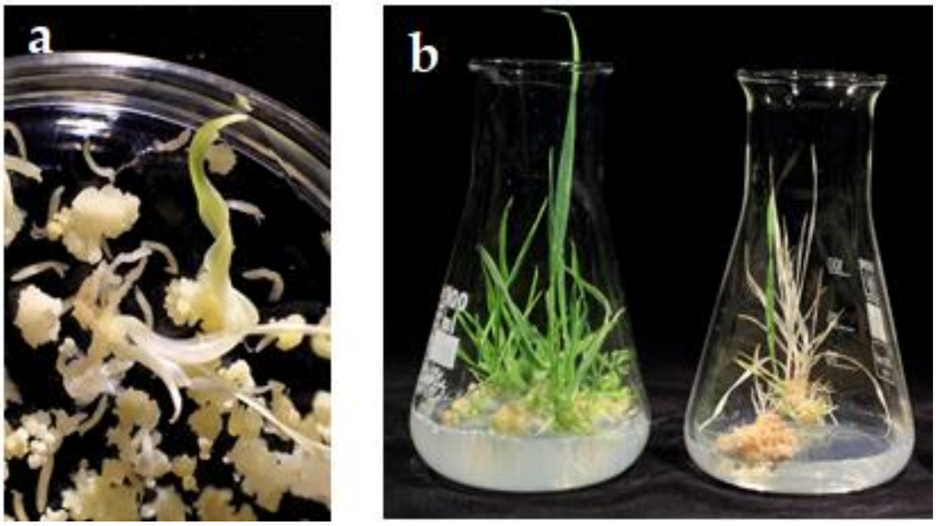

Figure S2. Induction and regeneration processes in barley anther culture, (**a**): pollen embryos or calli on induction medium after 4-5 weeks, (**b**): regenerating green and albinotic plantlets after 3 weeks on regeneration medium.

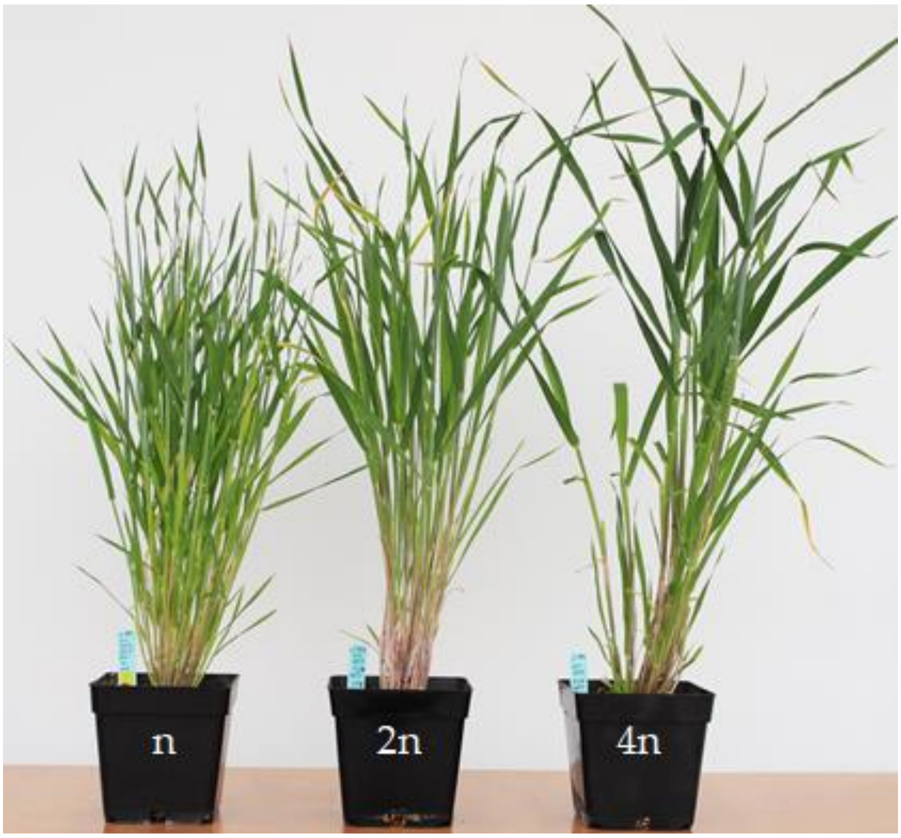

Figure S3. Regenerated plants from anthers of transgenic barley T1 generation, transgene Lim. The difference in appearance between a haploid plant (n), dihaploid plant (2n) and tetrahaploid plant (4n) are shown.

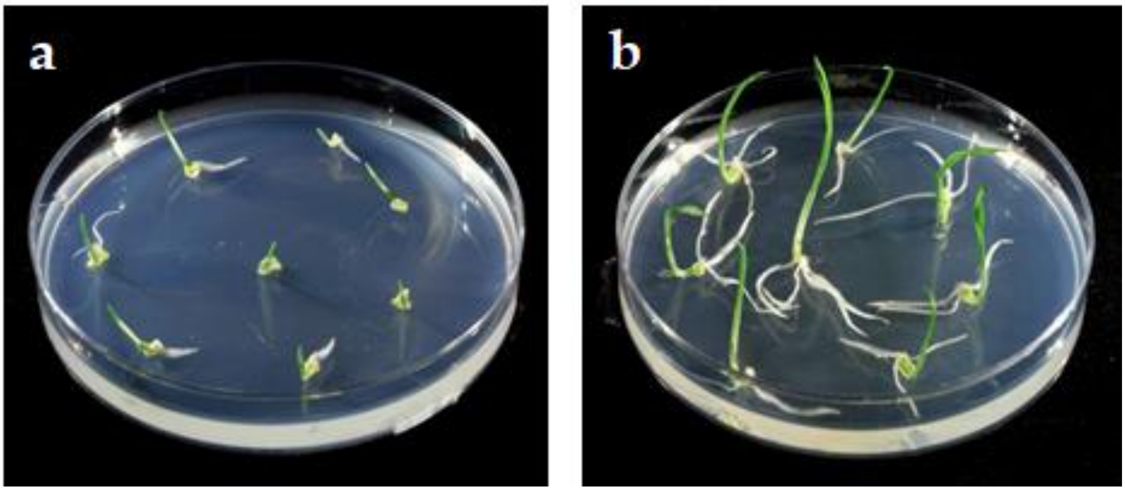

Figure S4. Transgenic barley embryo culture, (a): 5 days after cultivation, (b): 10 days after cultivation.

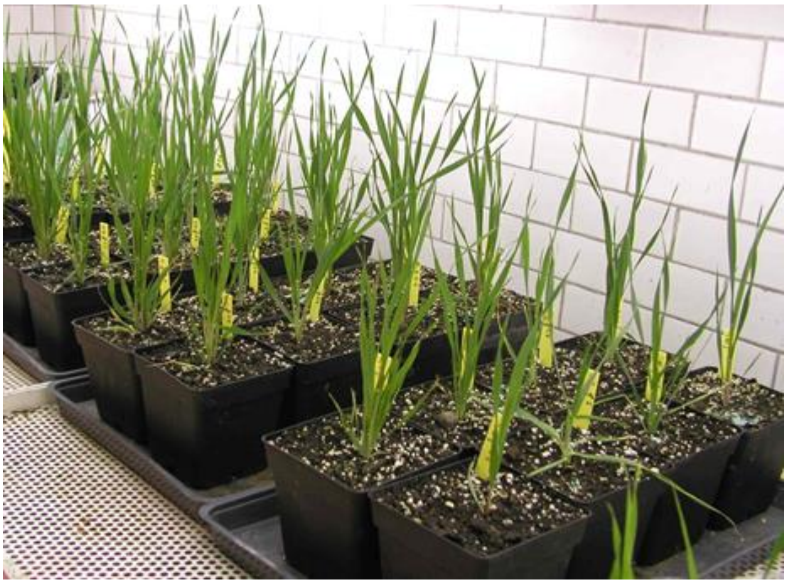

Figure S5. Transgenic plants (DH1 generation) regenerated from anther cultures.
